# Central nervous system infections in postnerosurgical patients

**DOI:** 10.1186/1753-6561-5-S6-P191

**Published:** 2011-06-29

**Authors:** HL Albornoz, L Ibias, A Gadea, F Porcires, R Brinckhaus, N Ramos, H Bagnulo

**Affiliations:** 1 Intensive Care Unit, Hospital Maciel, Montevideo, Uruguay; 2 Infection Control Commitee, Hospital Maciel, Montevideo, Uruguay

## Introduction / objectives

Central nervous system infections in neurosurgical patients (pts) are a seriuos complication with high morbidity and mortality. We describe characteristics of patients and episodes, microorganisms and evolution of neurosurgical pts with meningitis (M) or ventriculitis (V) in one ICU in Uruguay.

## Methods

Retrospective analysis of neurosurgical pts with M or V in a ten year period (2000-2010). M and V was defined based in cerebrospinal fluid findings (glucose <0.4 g/L, < 40% plasmatic glucose, leucocytes > 50/ mL (>50% neutrophils), lactate > 4 mM/L) and culture (definitive episodes). V required intraventricular procedure or device implantation.

## Results

69 pts (47 years, male 69%, SAPS II 33, mechanical ventilation 92%) developed 77 episodes (M 44, V 32). Neurosurgical diseases were trauma (39%), meningeal hemorrhage (20%), intracerebral hemorrhage (17%), intracranial tumor (12%). Cerebrospinal fluid leakage was present in 25%, ventriculostomy in 35% (catheter permanence 6.2 days), subdural catheter in 30% (catheter permanence 4.2 days). Microorganisms were mainly Gram negative bacilli (Acinetobacter sp (20, 26%), Klebsiella sp (7, 9%), Ps aeruginosa (7, 9%), Proteus sp (3, 3.9%), Enterobacter sp (3, 3.9%), S aureus (8, 10.4%), S coagulase negative (6, 7.8%), Enterococcus sp (3, 3.9%), Candida sp (5, 6.5%)). Crude mortality was 29% (20/69).

## Conclusion

In a selected group of seriously ill and high risk neurosurgical patients M and V were mainly caused for Gram negative bacilli and had high mortality.

## Disclosure of interest

None declared.

